# Dual-Directional Immunomodulatory Effects of Corbrin Capsule on Autoimmune Thyroid Diseases

**DOI:** 10.1155/2016/1360386

**Published:** 2016-09-18

**Authors:** Tianyi He, Ruxing Zhao, Yiran Lu, Wenjuan Li, Xinguo Hou, Yu Sun, Ming Dong, Li Chen

**Affiliations:** ^1^Department of Endocrinology, Qilu Hospital, Shandong University, Jinan, Shandong 250012, China; ^2^Institute of Endocrinology and Metabolism, Shandong University, Jinan, Shandong 250012, China

## Abstract

*Purpose.* To investigate the effects of Corbrin Capsule (CS-C-Q80), a drug derived from* Cordyceps sinensis *(Berk.) Sacc., on autoimmune thyroid diseases (AITD).* Methods.* 44 Patients with Graves's disease (GD) and 56 patients with Hashimoto's thyroiditis (HT) were randomly assigned to treatment group (GD-Tx and HT-Tx) or control group (GD-Ct and HT-Ct). The control groups were given methimazole or levothyroxine only while the treatment groups were given Corbrin Capsule (2.0 g tid) besides the same conventional prescriptions as control groups. Thyroid hormones, thyroid antibodies, and T lymphocyte subsets were quantified at baseline and 24 weeks posttreatment.* Results.* Significant drop of serum anti-TPO-Ab levels was observed in both GD-Tx and HT-Tx groups. Before treatment, GD patients had higher helper T cells compared to cytotoxic T cells, while HT patients suffered from a nearly inverted proportion of helper T/cytotoxic T cells. There was a significant drop of the helper T/cytotoxic T cells ratio in GD-Tx to the median of the normal ranges after Corbrin treatment for 24 weeks, while that in HT-Tx was elevated.* Conclusion.* Corbrin Capsule could restore the balance between helper T and cytotoxic T cells in both GD and HT patients with dual-directional immunomodulatory effects. And it could significantly reduce the autoantibody levels in both GD and HT.

## 1. Introduction

Autoimmune thyroid disease (AITD) is a typical organ-specific autoimmune disorder characterized by the elevated titer of autoantibodies and hyperactive T and B lymphocytes reactive to thyroid self-antigens [1]. AITD includes Graves' disease, Hashimoto's thyroiditis, and idiopathic hypothyroidism (atrophic Hashimoto's thyroiditis), Graves ophthalmopathy (GO), and postpartum thyroid dysfunction [2]. Among many AITDs, Graves' disease and Hashimoto's thyroiditis together have a prevalence of over 5% [3, 4], making autoimmunity to the thyroid gland the most common autoimmune disease affecting humans [5]. Currently, symptomatic treatments, including antithyroid drugs and thyroid hormone replacement are effective in controlling symptoms. But therapy targeting thyroid autoimmunity is lacking. Lack of intervention in the immunoinflammatory process would result in disease progression and high recurrence rate. In recent years, many studies have indicated that Corbrin Capsule (CS-C-Q80), a drug derived from the traditional Chinese medicine* Cordyceps sinensis* (CS) Sacc. [6], could exert immune-modulatory effects on many autoimmune-inflammatory diseases including lupus [7], chronic hepatitis [8], chronic kidney disease or kidney transplant [9, 10], asthma [11, 12], and diabetes mellitus [13, 14]. In China, some recent study reported that CS could have effects on reducing serum titer of anti-thyroid autoantibodies in AITD. Moreover, it was verified that CS could be effective on cell-mediated immunity in animal models of many diseases [15]. However, little is known of their influence on the hyperactive lymphocytes in autoimmune thyroid disease (AITD) in clinical practice. We made efforts to investigate the effects of Corbrin Capsule on the immune disturbance in AITD.

## 2. Materials and Methods

### 2.1. Subjects and Ethic Statement

Subjects were randomly selected from newly definitely diagnosed Graves' disease (GD) outpatients (*n* = 44) and Hashimoto's thyroiditis (HT) outpatients (*n* = 56) in the Department of Endocrinology, Qilu Hospital, between Apr 2013 and Aug 2014. A detailed clinical record was kept for each subject including history of disease and physical and laboratory examinations.

Exclusion criteria included unclear diagnosis of autoimmune thyroid diseases (AITDs) or any clues of other autoimmune disorders, recent infections, tumors, fever of any causes, and significantly elevated erythrocyte sedimentation rate. All donors did not receive other immunosuppressive or immunomodulatory drugs for at least 6 months upon sampling and during our treatment. Patients with recurrent Graves before recruitment were not excluded.

Our research was carried out in accordance with the Declaration of Helsinki (2008) of the World Medical Association. This study was approved by the Medical Ethical Committee of Qilu Hospital, Shandong University. Each patient signed a consent document. Baseline demographic parameters are summarized in Table 2.

### 2.2. Study Design

This study was conducted in a blinded manner. 44 patients with Graves' disease (GD) and 56 patients with hypothyroid Hashimoto's thyroiditis (HT) were randomly assigned into treatment groups (GD-Tx, *n* = 28, and HT-Tx, *n* = 39) or control groups (GD-Ct, *n* = 16, and HT-Ct, *n* = 17). The control groups were given conventional antithyroid drugs (for GD) or levothyroxine (for HT) only, while the treatment groups (GD-Tx and HT-Tx) were given Corbrin Capsule (2.0 g tid) besides the conventional prescriptions.

Each patient was followed up for at least 24 weeks. During treatment, necessary adjustments of the antithyroid drugs (for GD) or levothyroxine (for HT) doses were given by professional endocrinologist at each visit.

### 2.3. The Synopsis of Corbrin Capsule

The main composition of Corbrin Capsule (CS-C-Q80) is fermented powders of* Cordyceps sinensis* (ascomycetes). Cordyceps is an insect parasitizing fungus and being used as a traditional Chinese herb for more than 2,000 years. The active constituents of* C. sinensis* include cordycepin, polysaccharide, cordycepic acid, nucleosides, ergosterol, aminophenol, and trace elements. The therapeutic applications centered primarily on the key effects of increased oxygen utilization of ATP production and the stabilization of blood sugar metabolism as well. In previous views, its function of immunity regulation plays an important role in the antitumor effects, organ transplantation, and the prevention of kidney, liver, and heart disease [6].

### 2.4. Determination of Immunological Parameters

Fasting peripheral blood samples were obtained from each donor at 7 to 8 a.m. All samples were operated on within 2 hours after collection. Serum levels of thyroid hormones (including free T3, free T4, and thyroid stimulating hormone (TSH)) and the thyroid related autoantibodies (including TR-Ab, anti-TG-Ab, and anti-TPO-Ab) were determined by chemiluminescence immunoassay. The peripheral frequencies of CD3^+^ (total T) cells and CD3^+^CD4^+^ (helper T) and CD3^+^CD8^+^ (cytotoxic T) cells were quantified by flow cytometry at each time of follow-up (baseline, 4 weeks, 12 weeks, and 24 weeks after treatment). All tests were conducted in the centralized standardized laboratory in Qilu Hospital of Shandong University. Normal ranges and abbreviation of all relative indexes were listed in Table 1.

### 2.5. Statistical Analysis

All data are presented as mean ± SD or median ± quartile according to data distribution. Differences of parameters between each group were determined by Student's *t*-test unless the data were apparently skewed-distributed. All tests were performed and figures were generated by SPSS 18.0 system. *P* value less than 0.05 was considered statistically significant.

## 3. Results

### 3.1. Baseline Characteristics of Subjects

44 patients with Graves' disease (GD) and 56 patients with hypothyroid Hashimoto's thyroiditis (HT) were randomly assigned to treatment groups (GD-Tx and HT-Tx) or control groups (GD-Ct and HT-Ct), respectively. The average age of patients included in this study was 36.2 ± 14.19 versus 37.24 ± 10.26 years in GD-Tx and GD-Ct group and 41.40 ± 13.76 versus 43.82 ± 12.48 years in HT-Tx and HT-Ct group, respectively. The baseline demographic data is given in Table 2. Comparing the treatment and control groups of GD and HT, respectively, there were no significant differences between body mass index (BMI), blood pressure (BP), thyroid function, levels of thyroid related autoantibodies, and T lymphocyte populations (*P* > 0.05).

### 3.2. Therapeutic Effect of CS on GD Patients

**Figure 1:**
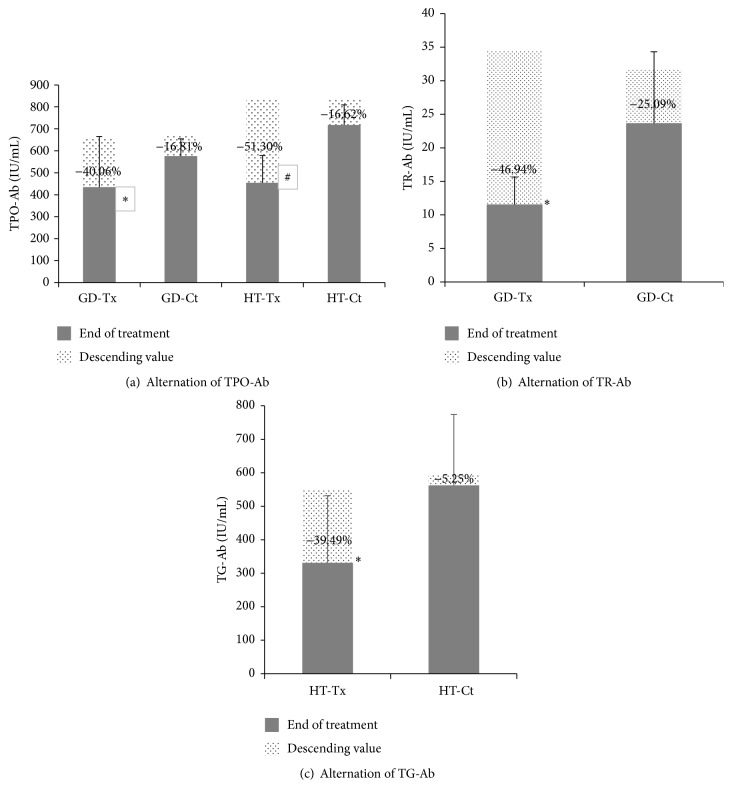
Alternations in peripheral autoantibody levels in AITD patients. Alternations in peripheral different kinds of thyroid related autoantibodies in treatment groups (GD-Tx and HT-Tx) and control groups (GD-Ct and HT-Ct), respectively. (a) Thyroid peroxidase antibody (TPO-Ab). ^*∗*^ *P* = 0.020, compared with GD-Ct; ^#^ *P* = 0.003, compared with HT-Ct. (b) Thyrotropin receptor antibody (TR-Ab). ^*∗*^ *P* < 0.001, compared with GD-Ct. (c) Thyroglobulin antibody (TG-Ab). ^*∗*^ *P* = 0.037, compared with HT-Ct.

At the end of treatment between GD-Tx and GD-Ct groups, the mean serum FT3 level (5.02 ± 3.55 versus 6.10 ± 1.18 pmol/L, *P* = 0.954) and FT4 levels (14.90 ± 2.13 versus 18.52 ± 2.73 pmol/L, *P* = 0.746) and median serum TSH level (1.34 ± 1.16 versus 1.14 ± 1.07, *P* = 0.741) showed no difference. The autoimmune data in GD-Tx group were given in Table 3. There were an increase of CD8^+^ (increased by 13.5%, *P* = 0.047), a decrease of CD4^+^ (decreased by 8.98%, *P* = 0.044), and a significant decrease in CD4/CD8 ratio (*P* = 0.022). Moreover, TPO-Ab and TR-Ab declined by 40.06% and 46.94% of baseline, respectively (*P* = 0.012 and *P* < 0.001) (Figure 1).

### 3.3. Therapeutic Effect of CS on HT Patients

Between HT-Tx and HT-Ct groups, thyroid functions were improved compared to the baseline at the end of treatment. Neither the mean serum FT4 levels (14.91 ± 2.14 versus 14.33 ± 1.78 pmol/L, *P* = 0.805) nor serum TSH level (3.36 ± 1.19 versus 3.09 ± 2.07, *P* = 0.386) showed statistical difference with the control group. However, TPO-Ab and TG-Ab declined by 51.30% and 39.49% of baseline, respectively (*P* = 0.022 and *P* = 0.037 compared with HT-Ct) (Figure 1). The autoimmune data in HT-Tx group were given in Table 4. There were a decrease in total CD3^+^ cells (decreased by 7.82%, *P* = 0.051), an increase of CD4^+^ (*P* = 0.791), a pronounced decrease of CD8^+^ (decreased by 17.7%, *P* = 0.031), and a relative increase of CD4/CD8 ratio (*P* = 0.024) after CS treatment.

As normal or hypothyroid HT were all included, we further divided the HT-Tx group into two groups, namely, euthyroidism HT-Tx group and hypothyroid HT-Tx group (data in supplement table in Supplementary Material available online at http://dx.doi.org/10.1155/2016/1360386). It was found that the decrease of TPO-Ab in euthyroidism HT-Tx group and the decrease of TG-Ab in hypothyroidism HT-Tx were more obvious, respectively. As for the CD4/CD8 ratio, the therapeutic effect of* Cordyceps sinensis* Sacc. was more significant on the hypothyroidism HT patients.

### 3.4. Cross-Sectional Comparison of Treatment and Control Group during 24 Weeks of Treatment

**Figure 2:**
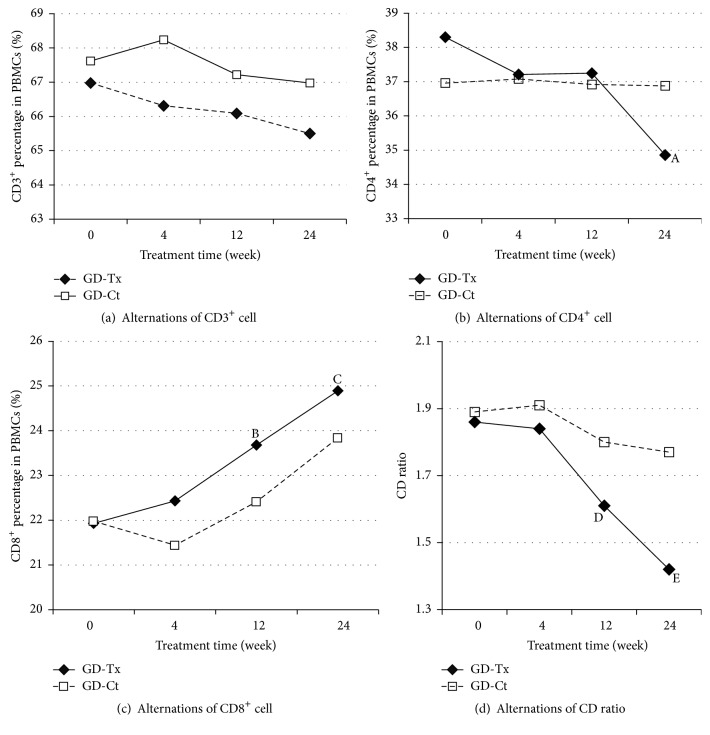
Alternations of peripheral T cell subsets along with treatment time in Graves' disease groups. Alternations in peripheral different kinds of T cell subsets in treatment groups (GD-Tx) and control groups (GD-Ct) of Graves disease. Four pictures are shown as follows: (a) CD3^+^ percentage in PBMCs (%), (b) CD4^+^ percentage in PBMCs (%), ^A^ *P* = 0.044, (c) CD8^+^ percentage in PBMCs (%), ^B^ *P* = 0.051 and ^C^ *P* = 0.047, and (d) CD4^+^/CD8^+^ ratio, ^D^ *P* = 0.047 and ^E^ *P* = 0.022.

**Figure 3:**
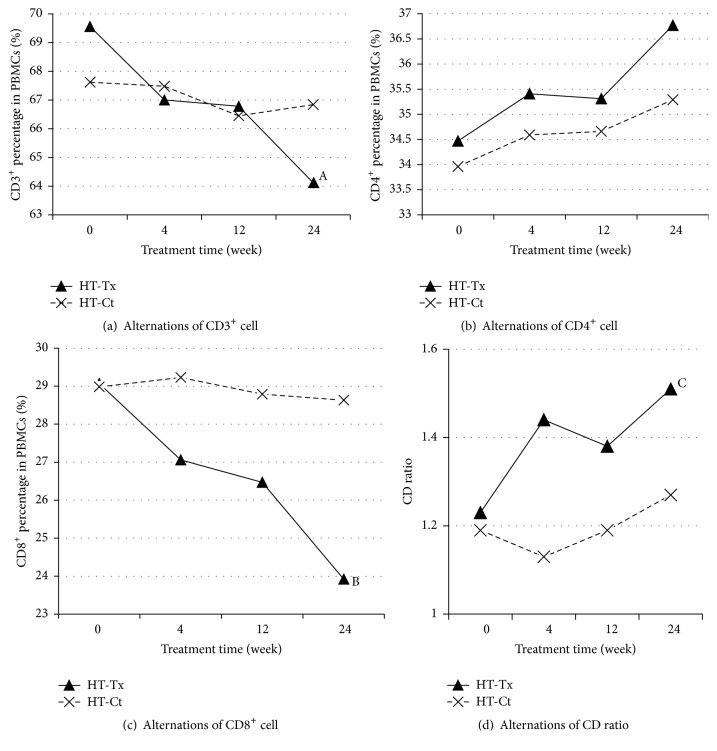
Alternations of peripheral T cell subsets along with treatment time in Hashimoto's thyroiditis (HT) groups. Alternations in peripheral different kinds of T cell subsets in treatment group (HT-Tx) and control group (HT-Ct) of Graves disease. Four pictures are, namely, in turn (a) CD3^+^ percentage in PBMCs (%), ^A^ *P* = 0.051, (b) CD4^+^ percentage in PBMCs (%), (c) CD8^+^ percentage in PBMCs (%), ^B^ *P* = 0.031, and (d) CD4^+^/CD8^+^ ratio, ^C^ *P* = 0.024.

Generally, there was a significant tendency of immune parameter changes in the longitudinal follow-up in Corbrin treatment groups compared to those in control groups (Figures 2 and 3). The comparison, respectively, between GD-Tx and GD-Ct and HT-Tx and HT-Ct group revealed the beneficial effect of combined therapy of* Cordyceps sinensis* Sacc. with conventional therapy (Tables 3 and 4).

## 4. Discussion

There are many organ-specific autoimmune disorders including autoimmune thyroid disease (AITD) [4], autoimmune arthritis [16], insulin-dependent diabetes mellitus [17], and myasthenia gravis [18] which is the most common disease in the clinical practice. AITD could lead to both hypothyroidism and hyperthyroidism. Graves' disease (GD) is usually the principal culprit for hyperthyroidism, whereas Hashimoto's thyroiditis (HT) is responsible for most cases of hypothyroidism in AITD. Although the detailed mechanisms for AITD largely remain unknown, the similar immune-mediated mechanisms of disease including lymphocytes hyperactivation and infiltration are the key processes in the development of both GD and HT [5]. Currently, interest in T lymphocyte subsets has arisen because of their critical role in regulation of the autoimmune process. CD3^+^CD4^+^ and CD3^+^CD8^+^ cells are the two major subsets in CD3^+^ lymphocytes (namely, T cells). CD3^+^CD4^+^ cells are traditionally called T helper cells since they assist other white blood cells in immunologic processes, including maturation of B cells into plasma cells and activation of CD3^+^CD8^+^ (namely, cytotoxic/suppressor T) cells. In both the entities of AITD, there is a broken balance between the T cell subsets. In fact, it has been suggested for more than two decades that T lymphocytes play different roles in the pathogenesis of the two major archetypes in human AITD [19]. A decrease in the CD4/CD8 ratio in hypothyroid Hashimoto's thyroiditis patients has long been observed, in contrast to the increase in the ratio in Graves' hyperthyroidism [20]. There was a decrease in CD8^+^ (suppressor/cytotoxic) T cells in the peripheral blood of patients with hyperthyroid Graves' disease [21]. And no cytotoxic effect of T cytotoxic/suppressor CD8^+^ cells on thyrocytes was observed in Graves' disease [22]. Actually, the pathogenic factors in Graves' hyperthyroidism are the autoaggressive CD4^+^ cells which drive the maturation of the plasma cells and secretion of stimulatory autoantibody targeting thyrotrophin (TSH) receptor (i.e., TR-Ab). In hypothyroid HT, however, the downregulated or even inverted CD4/CD8 ratio results from increased frequency of CD8^+^ cells, whose infiltration and cytolytic effects could be directly responsible for the destruction of the follicular thyroid and thyrocytes [23]. Thus, the divergence of T cell polarization may exactly explain for the main reason for the completely opposite clinical manifestations in GD and HT.

*Cordyceps sinensis* (CS) is an entomogenous fungus used as a tonic food and Chinese medicine to replenish health [6, 24]. Corbrin Capsule is a representative drug of* Cordyceps sinensis* (CS) Sacc. The biologic active component of Corbrin Capsule is 100% fermented powder of* Cordyceps sinensis *(CS-C-Q80) [25]. Corbrin Capsule has been reported to have immunomodulatory effects on many autoimmune-inflammatory diseases including cerebral disease [26] (in rat), type 1 diabetes, asthma, chronic obstructive pulmonary disease [27], and chronic nephritic disease [9]. In China, some recent studies reported that CS could significantly reduce serum titer of anti-thyroid autoantibody in both GD and HT. Moreover, it was verified that CS could be effective on cell-mediated immunity in animal models of many diseases [15]. However, there is no evidence on the immunomodulatory effects of CS-C-Q80 on T cell polarization in AITD patients. Since the divergence in T cell polarization in GD and hypothyroid HT patients plays an important role in the outcome of AITD, we investigated the effect of Corbrin Capsule on the number and distribution of peripheral blood T lymphocyte subsets in patients with GD and HT.

It had been observed for a long time that there was a reduction in the suppressor T cell subset and increase in the helper T cell subset in peripheral blood from GD patients [28]. Further study suggested that the decreases in the percentage of CD8^+^ cells (cytotoxic/suppressor cell) are associated with abnormalities in the immune system [29]. Aust et al. revealed that CD8^+^ cells, as well as NK cells, may help prevent pathogenic B cell infiltration of the thyroid [30]. It is well documented that proinflammatory Th1-type cytokines, including tumor necrosis factor-*α* (TNF-*α*), IFN-*γ*, and IL-2, play pivotal roles in the pathogenesis of GD in the active state [25–27]. Th2 rather than Th1 response is in the domination nonactive state. The Th2 dominance subsequently facilitates the T cell-dependent B cell activation and the secretion of stimulatory autoantibody, TS-Ab, from mature pathogenic B (plasma) cells into blood, leading to hyperactive thyrocyte functions and hyperthyroxinemia [31]. Since CD8^+^ cells can work to counteract Th2 dominance, suppression of CD8^+^ cells by this Th2 response may accentuate the symptoms of GD. Moreover, the increase of CD4^+^ cells, which indicates hyperactivation of T helper subset, could also promote the maturation of pathogenic plasma cells from B lymphocyte, thus exacerbating the hyperthyroidism in GD. In this study, we observed a higher CD4^+^/CD8^+^ ratio in untreated GD patients, which was in accordance with previous publications. After treatment with antithyroid drugs combined with CS-C-Q80, the CD4^+^/CD8^+^ ratio returned to the median of normal ranges (*P* = 0.022) accompanied by a significant drop of TR-Ab and TPO-Ab levels. However, there were no significant changes in these immune parameters in GD-Ct before and after traditional treatment suggesting a suppressive effect of humoral autoimmune reaction of CS-C-Q80 on GD.

HT, also referred to as chronic lymphocytic thyroiditis, is an autoimmune disease in which the thyroid gland is attacked by a variety of cell- and antibody-mediated immune processes [32]. A decrease in the CD4/CD8 ratio in HT hypothyroid patients was observed [23], in contrast to an increase in the ratio in autoimmune hyperthyroid patients. This suggests a different role for T lymphocytes in the pathogenesis of the two major human autoimmune thyroid diseases [22, 23]. The anti-TG and anti-TPO autoantibodies are usually seen elevated in HT, which is responsible for the subsequent antibody-mediated immune processes. Although a small percentage of patients may have nondetectable antibodies present, cell-mediated cytotoxicity is a substantial factor behind the apoptotic fall-out in HT. Activation of cytotoxic T (CD3^+^CD8^+^) cells in response to cell-mediated immune response affected by T helper (CD3^+^CD4^+^) cells is central to thyrocyte destruction. CD8^+^ T cells have cytotoxic properties in Hashimoto's thyroiditis [23] and can induce thyroid epithelial cell hyperplasia and fibrosis [33]. These indicate that clonal expansion of CD8^+^ T cells in HT can be detected in peripheral blood and support the role of CD8^+^ T cells in cell-mediated autoimmune attacks on the thyroid gland in HT [34]. During the natural course of HT, most patients are euthyroid at the beginning while half of them develop hypothyroidism during long-term follow-up [35]. Few of them suffered from a period of hyperthyroidism mostly due to destruction of follicular thyroid and release of thyroid hormones as what happened in other types of thyroiditis mostly among children. The latter usually goes undetected as it is transient, lasting only a few months [32]. Thus the natural course of HT is attributed to and reflects the different stages of autoimmune process targeting thyroid. Conventional treatment of L-thyroxine could efficiently restore euthyroidism after 12 weeks but without the drop of antibodies. Though about 8% of these patients maintain a normal thyroid function after withdrawal of L-thyroxine replacement for no significant change in CD8^+^ percentages, CD8^+^ percentages in most HT patients are higher and could cause a chronic nature. In this study, we focused mostly on HT patients without hyperthyroid. All these patients had a significantly elevated anti-TPO-Ab and anti-TG-Ab levels and impaired thyroid function (TSH) which were characterized by lower CD4^+^ T cells and higher CD8^+^ T cells as seen in Table 2. With the treatment of Corbrin Capsule, there was a significant drop in anti-TPO-Ab level and total CD3^+^ cells and CD8^+^ T cells. The increased CD4^+^/CD8^+^ ratio (*P* = 0.024) indicates that CS-C-Q80 could correct the cellular immune disorder, at least partially, by inhibiting CD8^+^ expansion.

Of note, a number of studies have shown that CS can positively stimulate the activation of T lymphocytes, B lymphocytes, natural killer cells, and macrophages [36]. In our study, we found that CS-C-Q80 could also inhibit the proliferation of CD8^+^ T cells in cytotoxic T-dominant autoimmune status or CD4^+^ T cells in helper T-dominant (especially Th2-dominant) autoimmune status and restore the helper T/cytotoxic T cells balance to the median value of normal ranges. These results suggest a bidirectional immunomodulatory effect of CS under different immunological and inflammatory context [37].

## 5. Conclusion

GD and HT patients have different pathological changes in helper T/cytotoxic T cells polarization. CS-C-Q80 could restore the balance between helper T and cytotoxic T cells in both GD and HT patients with dual-directional immunomodulatory effects. In addition, it could significantly reduce the autoantibody levels in both GD and HT and thus could attenuate the immune disturbance in both types of AITD. Since the immune disturbance is considered the culprit of the recurrence for GD and to be of a chronic nature for typical HT, the long-term effects of CS-C-Q80 on preventing relapse of AITD are warranted in the near future.

## Supplementary Material

Among HT-Tx group, euthyroidism patients and hypothyroidism patients were compared. The decrease of TPO-Ab in euthyroidism HT-Tx group and the decrease of TG-Ab in hypothyroidism HT-Tx group were more obvious, respectively. As for the CD4/CD8 ratio, hypothyroidism HT-Tx restored nearer to the median of normal ranges other than euthyroidism HT.

## Figures and Tables

**Table 1:** Abbreviations and normal ranges.

Abbreviation	Full name	Normal ref. range	Unit
fT3	Free triiodothyronine	2.3–6.3	pmol/L
fT4	Free tetraiodothyronine	10.3–24.5	pmol/L
TSH	Thyroid stimulating hormone	0.35–5.5	UIU/mL
TPO-Ab	Anti-thyroid peroxidase antibody	0–60	U/mL
TR-Ab	Thyrotrophin receptor antibody	0.01–30	IU/mL
TG-Ab	Antithyroglobulin antibodies	0–4.11	IU/mL
CD3	Cluster of differentiation 3	55–80	%
CD4	Cluster of differentiation 4	25–45	%
CD8	Cluster of differentiation 8	15–35	%
CD4/CD8	Ratio of CD4 and CD8	1.0–2.0	—

The abbreviations and normal ranges of all indexes. All the reference ranges are from the endocrinology laboratory and immunology laboratory in Qilu Hospital among Chinese population.

**Table 2:** Baseline characteristics of subjects.

	GD			HT		
	Tx (*n* = 28)	Ct (*n* = 16)	*P* value	Tx (*n* = 39)	Ct (*n* = 17)	*P* value
Age (year)	36.2 ± 14.19	37.24 ± 10.26	0.156	41.40 ± 13.76	43.82 ± 12.48	0.168
Gender (male/female)	14/28	6/16	0.492	11/39	7/17	0.254
BMI (kg/m^2^)	21.11 ± 2.46	22.94 ± 3.16	0.061	23.13 ± 3.57	22.83 ± 4.01	0.071
SBP (mmHg)	117.87 ± 12.26	114.55 ± 11.43	0.112	120.25 ± 15.24	114.27 ± 17.64	0.057
DBP (mmHg)	76.47 ± 10.23	78.58 ± 9.15	0.135	86.08 ± 13.44	77.98 ± 10.21	0.106
HR (/min)	88.53 ± 5.82	86.89 ± 4.53	0.171	75.42 ± 9.32	78.36 ± 8.34	0.770
FT3 (pmol/L)	9.52 ± 7.53	8.96 ± 1.43	0.308	3.39 ± 1.01	3.42 ± 1.12	0.640
FT4 (pmol/L)	23.01 ± 17.26	25.93 ± 10.46	0.112	11.83 ± 3.64	10.47 ± 3.59	0.318
TSH (*μ*IU/L)	0.07 ± 0.13	0.09 ± 0.17	0.451	10.94 ± 6.52	9.75 ± 7.21	0.522
TPO-Ab (IU/mL)	659.28 ± 585.89	672.39 ± 463.51	0.086	820.51 ± 372.45	829.39 ± 388.29	0.455
TG-Ab (IU/mL)	—	—	—	547.8 ± 331.45	593.28 ± 273.65	0.383
TR-Ab (IU/mL)	34.51 ± 17.97	31.16 ± 11.46	0.128	—	—	—
CD3^+^ (%)	66.98 ± 9.40	67.62 ± 8.36	0.196	69.56 ± 8.57	67.62 ± 8.36	0.146
CD4^+^ (%)	38.30 ± 5.37	36.96 ± 2.29	0.227	34.47 ± 6.74	33.96 ± 4.29	0.238
CD8^+^ (%)	21.93 ± 4.46	21.98 ± 6.56	0.348	29.07 ± 7.06	28.98 ± 6.54	0.074
CD4^+^/CD8^+^ ratio	1.86 ± 0.66	1.89 ± 0.79	0.105	1.23 ± 0.55	1.19 ± 0.68	0.089

44 Graves disease (GD) outpatients and 56 hypothyroid Hashimoto's thyroiditis (HT) outpatients were newly definitely diagnosed and enrolled. All general data and thyroid hormones and related antibodies and cell immune indicators were listed. GD-Tx: GD treatment group; GD-Ct: GD control group; HT-Tx: HT treatment group; and HT-Ct: HT control group.

**Table 3:** Baseline and end of treatment of subjects in Graves' disease.

	GD-Tx			GD-Ct		
	Baseline	End of treatment	*P* value	Baseline	End of treatment	*P* value
FT3 (pmol/L)	9.52 ± 7.53	5.02 ± 3.55	0.098	8.96 ± 1.43	6.10 ± 1.18	0.077
FT4 (pmol/L)	23.01 ± 17.26	14.90 ± 2.13	0.130	25.93 ± 10.46	18.52 ± 2.73	0.340
TSH (*μ*IU/L)	0.07 ± 0.13	1.34 ± 1.16	0.042	0.09 ± 0.17	1.14 ± 1.07	<0.001
TPO-Ab (IU/mL)	659.28 ± 585.85	434.99 ± 229.93	0.012	672.39 ± 463.51	575.64 ± 361.87	0.313
TR-Ab (IU/mL)	34.51 ± 17.97	11.57 ± 4.07	<0.001	31.16 ± 11.46	27.07 ± 8.14	0.083
CD3^+^ (%)	66.98 ± 9.40	66.31 ± 9.70	0.462	67.62 ± 8.36	66.98 ± 5.31	0.761
CD4^+^ (%)	38.30 ± 5.37	34.86 ± 2.03	0.044	36.96 ± 2.29	36.88 ± 3.46	0.947
CD8^+^ (%)	21.93 ± 4.46	24.89 ± 3.29	0.047	21.98 ± 6.56	23.84 ± 5.12	0.150
CD4/CD8 ratio	1.86 ± 0.66	1.42 ± 0.19	0.022	1.89 ± 0.79	1.60 ± 0.43	0.209

The data of GD-Tx group and GD-Ct group were compared between the start and end of treatment (24 weeks). In the GD-Tx groups, there were a significant decrease of CD4^+^, an increase of CD8^+^, and a decreased CD4/CD8 ratio compared to the baseline. None of the parameters changed significantly in GD-Ct group.

**Table 4:** Baseline and end of treatment of subjects in Hashimoto's thyroiditis.

	HT-Tx			HT-Ct		
	Baseline	End of treatment	*P* value	Baseline	End of treatment	*P* value
FT3 (pmol/L)	3.39 ± 1.01	4.19 ± 1.03	0.469	3.42 ± 1.12	3.92 ± 1.30	0.754
FT4 (pmol/L)	11.83 ± 3.64	14.91 ± 2.14	0.002	10.47 ± 3.59	14.33 ± 1.78	0.003
TSH (*μ*IU/L)	10.94 ± 6.52	3.36 ± 1.19	0.003	9.75 ± 7.21	3.09 ± 2.07	0.031
TPO-Ab (IU/mL)	820.51 ± 572.4	454.05 ± 227.96	0.022	829.39 ± 388.29	717.19 ± 213.63	0.217
TG-Ab (IU/mL)	547.8 ± 331.45	331.45 ± 124.61	0.037	593.28 ± 273.65	561.82 ± 196.97	0.603
CD3^+^ (%)	69.56 ± 8.57	64.12 ± 8.18	0.051	67.62 ± 8.36	68.11 ± 2.55	0.838
CD4^+^ (%)	34.47 ± 6.74	36.77 ± 4.32	0.791	33.96 ± 4.29	34.00 ± 2.19	0.962
CD8^+^ (%)	29.07 ± 7.06	23.92 ± 6.20	0.031	28.98 ± 6.54	25.91 ± 3.17	0.295
CD4/CD8 ratio	1.23 ± 0.55	1.51 ± 0.46	0.024	1.19 ± 0.68	1.33 ± 0.21	0.457

The data of HT-Tx group and HT-Ct group were compared between the start and end of treatment (24 weeks). In the HT-Tx group, there were an increase of CD4^+^, a significant decrease of CD8^+^, and an increased CD4/CD8 ratio compared to the baseline. None of the parameters changed significantly in HT-Ct group.
