# Supplementary material for: Dual-Directional Immunomodulatory Effects of Corbrin Capsule on Autoimmune Thyroid Diseases
Source: Evid Based Complement Alternat Med. 2016 Sep 18;2016:1360386. doi: 10.1155/2016/1360386 (PMC5045992; doi:10.1155/2016/1360386)
Supplement: Supplementary file 1 — Among HT-Tx group, euthyroidism patients and hypothyroidism patients were compared. The decrease of TPO-Ab in euthyroidism HT-Tx group and the decrease of TG-Ab in hypothyroidism HT-Tx group were more obvious, respectively. As for the CD4/CD8 ratio, hypothyroidism HT-Tx restored nearer to the median of normal ranges other than euthyroidism HT. [file 1360386.f1.pdf]

Supplement Table Therapeutic effect of Cordyceps sinensis sacs in normal HT-Tx and hypothyroidism HT-Tx (24 weeks treatment)

	Euthyroidism HT-Tx		P value	Hypothyroidism HT-Tx		P value
	Baseline	End of treatment		Baseline	End of treatment	
FT3 (pmol/L)	4.24±0.47	3.99±0.64	0.179	3.74±1.04	4.39±1.33	0.222
FT4 (pmol/L)	13.13±0.88	14.05±2.17	0.491	10.24±2.49	15.77±1.83	<0.001
TSH (uIU/L)	3.39±0.37	3.08±0.77	0.307	18.01±7.77	3.63±1.49	<0.001
TPO-Ab (IU/ml)	905.7±592.3	592.55±156.65	0.011	813.14±482.15	511.87±292.44	0.052
TG-Ab (IU/ml)	262.7±162.5	239.23±128.22	0.767	832.91±483.8	312.56±144.56	0.043
CD3+ (%)	73.27±6.7	68.63±4.9	0.072	65.86±8.96	63.62±8.20	0.390
CD4+ (%)	38.04±7.69	35.35±4.17	0.331	33.6±5.11	35.34±5.28	0.271
CD8+ (%)	31.27±7.64	27.00±4.03	0.079	26.86±6.06	23.84±7.93	0.032
CD4/CD8 ratio	1.30±0.61	1.38±0.28	0.963	1.21±0.53	1.57±0.58	0.010

Supplement Table Among HT-Tx group, euthyroidism patients and hypothyroidism patients were compared. The decrease of TPO-Ab in euthyroidism HT-Tx group and the decrease of TG-Ab in hypothyroidism HT-Tx group were more obvious, respectively. As for the CD4/CD8 ratio, hypothyroidism HT-Tx restored nearer to the median of normal ranges other than euthyroidism HT.
